# Enhanced invasion and survival of antibiotic- resistant *Klebsiella pneumoniae* pathotypes in host cells and strain-specific replication in blood

**DOI:** 10.3389/fcimb.2025.1522573

**Published:** 2025-02-14

**Authors:** Kathleen Klaper, Yvonne Pfeifer, Lena Heinrich, Marcel Prax, Oleg Krut, Isabelle Bekeredjian-Ding, Anika Wahl, Martin A. Fischer, Heike Kaspar, Stefan Borgmann, Roman G. Gerlach, Guido Werner

**Affiliations:** ^1^ Department of Infectious Diseases, Robert Koch Institute, Wernigerode, Germany; ^2^ Department of Infectious Diseases, Robert Koch Institute, Berlin, Germany; ^3^ Division of Microbiology, Paul Ehrlich Institute, Langen, Germany; ^4^ Division of Antibiotic Resistance Monitoring, Federal Office of Consumer Protection and Food Safety, Berlin, Germany; ^5^ Department of Infectious Diseases and Infection Control, Hospital Ingolstadt, Ingolstadt, Germany

**Keywords:** adhesion, invasion, replication, *Klebsiella pneumoniae*, serum resistance, platelet concentrate, cell morphology, normoxia

## Abstract

**Background:**

*Klebsiella pneumoniae* is one of the most important opportunistic pathogens causing healthcare-associated and community-acquired infections worldwide. In recent years, the increase in antibiotic resistance and infections caused by hypervirulent *K. pneumoniae* poses great public health concerns. In this study, host-pathogen interactions of different *K. pneumoniae* strains of human and animal origins were analyzed in microbiological, cell-biological and immunological experiments.

**Methods:**

*In vitro* infection experiments using representatives of different *K. pneumoniae* pathotypes and various epithelial and macrophage cell lines were executed analyzing adhesion, invasion and intracellular replication. Experimental conditions involved normoxia and hypoxia. Furthermore, survival and growth of further *K. pneumoniae* isolates expressing defined siderophores in blood (platelet concentrates, serum) was investigated. All experiments were done in triplicate and statistically significant differences were determined.

**Results:**

Significant differences in adhesion and invasion capability, phagocytosis resistance and intracellular replication were measured between different *K. pneumoniae* pathotypes. Especially, ESBL-producing *K. pneumoniae* isolates demonstrated increased invasion in host cell lines and survival in macrophages. A strong cytotoxic effect on intestinal cells was observed for hypervirulent *K. pneumoniae*. The results from our investigations of the growth behavior of *K. pneumoniae* in platelets and serum showed that siderophores and/or an enlarged capsule are not essential factors for the proliferation of (hypervirulent) *K. pneumoniae* strains in blood components.

**Conclusion:**

Our *in vitro* experiments revealed new insights into the host-pathogen interactions of *K. pneumoniae* strains representing different pathovars and clonal lineages in different infectious contexts and hosts. While a clear limitation of our study is the limited strain set used for both infection and as potential host, the results are a further step for a better understanding of the pathogenicity of *K. pneumoniae* and its properties essential for different stages of colonization and infection. When developed further, these results may offer novel approaches for future therapeutics including novel “anti-virulence strategies”.

## Introduction

*Klebsiella pneumoniae* is a Gram-negative bacterium that has emerged as a significant human pathogen. It poses a serious threat to public health by causing a wide range of infections in humans, including pneumonia, urinary tract infections, and bloodstream infections (Rahi et al., 2024). Originally recognized for its role in causing pneumonia more than 100 years ago, this microorganism has recently gained renewed attention due to its ability to adapt and evolve, resulting in enhanced virulence and evasion of host immune responses (Lam et al., 2018b; Martin and Bachman, 2018). Central to the pathogenicity of *K. pneumoniae* is its polysaccharide capsule, a complex outer layer that shields the bacterium from host defenses and contributes to its survival in diverse environments (Domenico et al., 1994; Sahly et al., 2000; Opoku-Temeng et al., 2019).

In the past 20 years, a continuous increase of *K. pneumoniae* with resistance to cephalosporines and carbapenems has been detected in healthcare settings worldwide. Extended-spectrum beta-lactamases (ESBL) are enzymes that confer resistance to a broad range of beta-lactam antibiotics, including penicillins and first- to third-generation cephalosporins (Fostervold et al., 2022; Hawkey et al., 2022). The prevalence of ESBL-producing *K. pneumoniae* strains from invasive infections varies considerably. For instance, rates of invasive *K. pneumoniae* with ESBL phenotypes ranged among European countries in 2021 from <5% to >80% with 34% on average and an overall increasing trend in recent years (Anonymous, 2023). Carbapenems are one of the few remaining treatment options for ESBL-producing and other drug-resistant *Klebsiella* infections. Thus, the emergence of carbapenem resistance is of major concern because the treatment options are limited only to a few remaining substances (Isler et al., 2022; Ding et al., 2023). Carbapenem resistance in *K. pneumoniae* is often mediated by the acquisition of carbapenemases of various types including KPC, OXA, and NDM (Argimón et al., 2021; Gorrie et al., 2022; Ding et al., 2023).

Hypervirulence in *K. pneumoniae* is a phenomenon characterized by strains that are able to cause severe infections in healthy individuals, in contrast to the usual susceptibility of immune-compromised patients to bacterial infections by classical healthcare pathogens (Russo and Marr, 2019; Wyres et al., 2020; Russo et al., 2024). One defining feature of hypervirulent *K. pneumoniae* is hypermucoviscosity, attributed to an increased production of capsular polysaccharides. This hypermucoviscous phenotype is associated with specific virulence factors such as the presence and expression of the regulators of the mucoid phenotype *rmpA* and/or *rmpA2*, which finally enhances capsule production, providing protection against phagocytosis and contributing to the overall virulence of the bacterium (Russo and Marr, 2019; Wyres et al., 2020; Mendes et al., 2023; Russo et al., 2024).

The role of siderophores in the virulence of hypervirulent *K. pneumoniae* strains has attracted significant attention in recent studies (Russo et al., 2021; Kumar et al., 2024; Wahl et al., 2024). Siderophores are small molecules that help bacteria acquire iron, an essential nutrient for their survival and pathogenicity. *K. pneumoniae*, like many other pathogens, utilizes siderophores to scavenge iron from the host environment, thereby enhancing its ability to colonize the host and cause infection. These siderophores not only contribute to the bacterial iron acquisition but also play a significant role in modulating the host immune response, ultimately increasing the pathogenicity of the bacterium (Bachman et al., 2012; Holden et al., 2016; Russo et al., 2021; Mendes et al., 2023). Several types of siderophores have been identified including colibactin *clb*, yersiniabactin *ybt*, salmochelin *iro*, and aerobactin *iuc*, among others. Each siderophore type plays a distinct role in iron acquisition and may have different implications for the pathogenicity of *K. pneumoniae.* Kleborate, a tool widely used for the genomic characterization of *Klebsiella* isolates, assesses the virulence potential of *K. pneumoniae* strains based on the presence of specific siderophores and assigns a virulence score (Lam et al., 2021; Russo et al., 2024); whereas the tool Kaptive delineates the capsule and polysaccharide composition (Lam et al., 2022). Traditionally, hypervirulent *K. pneum*oniae were less resistant to antibiotics, making them susceptible to common treatments. However, the acquisition of ESBL or carbapenemase genes by these hypervirulent strains not only enhances their ability to cause severe disease but also limits therapeutic options tremendously (Wyres et al., 2020; Mendes et al., 2023; Merla et al., 2024; Wahl et al., 2024). Specific world regions are primarily affected by this converging effect, in other regions and countries these strains occur sporadically (Liao et al., 2020; Hu et al., 2024; Merla et al., 2024; Wahl et al., 2024; Zhou et al., 2024).

Only a few studies investigated the association between increased antimicrobial resistance and virulence in *K. pneumoniae* (Mendes et al., 2023). Sahley et al. described that the acquisition of resistance-encoding plasmids is associated with increased fimbrial expression and increased adhesion to epithelial cells (Sahly et al., 2008). Another study investigated pathophysiological differences between antibiotic-susceptible, classical *K. pneumoniae* (c*Kp*) and hypervirulent *K. pneumoniae* (hv*Kp*) and demonstrated that hv*Kp* resisted phagocyte-mediated clearance and replicated in mouse liver macrophages (Wanford et al., 2021). In the present study we follow previous observations and extend these analyses by providing new data about pathogen-host interactions of various *K. pneumoniae* strains of human and animal origin. Life-threatening *K. pneumoniae* infections include the capability of the pathogen to adhere to surfaces, to invade host cells and to translocate through cellular barriers. Therefore, we investigate adhesion, invasion and replication capacities to various human and animal cell lines as well as the phagocytosis resistance of c*Kp*, ESBL-producing *K. pneumoniae* (ESBL-c*Kp*) and hv*Kp* isolates. The cell assays were carried out under normoxia and hypoxia conditions, the latter to better reflect the natural habitat of the bacterium. Furthermore, the resistance and growth in platelet concentrates and in human sera were investigated mimicking the infectious lifecycle of *K. pneumoniae*. The aim was to identify potential host associations of *K. pneumoniae* and to reveal novel findings about host adaptation of different pathotypes contributing to their pathogenicity and survival under challenging immunological conditions.

## Materials and methods

### Bacterial isolates and antibiotic susceptibility testing

We used the following isolates of *K. pneumoniae* for most of the functional assays: 17-0683 and 17-0684, representing antibiotic-susceptible classical *K. pneumoniae* (c*Kp*); two ESBL-producing isolates (16-0382 and 16-0383; ESBL-c*Kp*) and two hypervirulent isolates (17-0609 and 18-0005; hv*Kp*). Isolates 17-0683 and 17-0684 were isolated from blood cultures and were susceptible to all antibiotics tested, except ampicillin. Ampicillin resistance is mediated by an SHV-type beta-lactamase that is characteristic of the *K. pneumoniae* species (Livermore, 1995). The ESBL-producing isolates 16-0382 and 16-0383 originated from an urine culture and a throat swab, respectively. Both isolates were resistant to third-generation cephalosporins and possessed the ESBL gene *bla*_CTX-M-15_. The two selected hv*Kp* isolates 17-0609 and 18-0005 belonged to the sequence types ST2398 and ST66, respectively (Klaper et al., 2021). Both isolates showed capsule type K2 and carried the virulence genes *ybt, iro, iuc, clb* and *rmpA*. Furthermore, these hv*Kp* isolates were hypermucoviscous, confirmed by a positive string test (Pichler et al., 2017; Wahl et al., 2024). Characteristics of the above mentioned six isolates and further isolates used to determine serum resistance and growth in platelet concentrates are given in **Table 1**. The bacterial species was determined by an automated system (Vitek 2 ID card, bioMérieux, Nuertingen, Germany); and antimicrobial susceptibility testing (ampicillin, cefotaxime, ceftazidime, meropenem) was done using broth microdilution with result interpretation according to the criteria of EUCAST version v14.0 (EUCAST, 2024).

**Table 1:** *Klebsiella pneumoniae* isolates used for this study (grouped by pathotype).

Designation	Origin	Beta-lactam resistances	Beta-lactamase*	MLST type	Capsule type	O-locus	Virulence genes*	String test	Virulence score*	Pathotype category	Reference
17-0683^1^	H: blood culture	AMP	SHV-11	ST1754	KL62	O1/O2v1	*-*	–	0	c*Kp*	This study
17-0684^1^	H: blood culture	AMP	SHV-108	ST889	KL54	O1/O2v1	*ybt*	–	1	c*Kp*	This study
18-0014	A: bovine mastitis	AMP	SHV-116	ST609	KL114	O1/O2v1	*-*	–	0	c*Kp*	This study
18-0018	A: bovine mastitis	AMP	SHV-11	ST403	KL23	O1/O2v2	*-*	+	0	c*Kp*	This study
18-0030^2^	A: bovine mastitis	AMP	SHV-116	ST310	KL1	O1/O2v1	*-*	–	0	c*Kp*	This study
ATCC 13883^2^	unknown	AMP	SHV-11	ST395	K3	O1/O2v1	*ybt,rmpA,clb*	–	2	c*Kp*	Reference strain
PEI-B-P-08^2^	H: invasive isolate	AMP	SHV-11	ST48	K62	O1/O2v1	*ybt*	–	1	c*Kp*	Reference strain (Spindler-Raffel et al., 2017)
16-0382^1^	H: urine sample	AMP, CTX, CAZ	CTX-M-15, SHV-28, OXA-1	ST15	KL24	O1/O2v1	*ybt*	–	1	ESBL-c*Kp*	(Becker et al., 2018)
16-0383^1^	H: throat swab	AMP, CTX, CAZ	CTX-M-15, SHV-83, OXA-1	ST29	KL30	O1/O2v2	*-*	+	0	ESBL-c*Kp*	(Becker et al., 2018)
18-0414^2^	H: unknown	AMP, CTX, CAZ, MER	SHV-11, CTX-M-15, OXA-48, OXA-1, TEM-1	ST392	K27	O4	*ybt,rmpA2*	–	1	ESBL-c*Kp*	(Wahl et al., 2024)
ATCC 700721^2^	H: sputum	AMP	SHV-11 + 12, OXA-9, TEM-1	ST38	K52	OL13	*-*	–	0	ESBL-c*Kp*	Reference strain
17-0609^1,2^	H: liver abscess	–	–	ST2398	KL2	O1/O2v2	*ybt,iro,iuc,clb,rmpA*	+	5	hv*Kp*	(Pichler et al., 2017)
18-0005^1^	H: throat swab	–	–	ST66	KL2	O1/O2v2	*ybt,iro,iuc,clb,rmpA*	+	5	hv*Kp*	(Klaper et al., 2021)
19-0213^2^	H: liver abscess	AMP, CTX, CAZ, MER	SHV-1, CTX-M-55, NDM-1, OXA-48	ST23	K57	O1/O2v1	*ybt,iuc,rmpA,rmpA2*	+	4	hv*Kp*	(Wahl et al., 2024)
17-0736^2^	H: blood culture	AMP	SHV-11	ST5	KL39	O1/O2v1	*ybt,iro,iuc,rmpA*	+	4	hv*Kp*	(Wahl et al., 2024)
19-0036^2^	H: blood culture	AMP	SHV-1	ST412	K57	O3b	*iro,iuc,rmpA,rmpA2*	–	3	hv*Kp*	(Wahl et al., 2024)
NCTC 14052^2^	H: liver abscess	AMP	SHV-11	ST23	KL1	O1/O2v2	*ybt,iro,iuc,clb,rmpA,rmpA2*	+	5	hv*Kp*	Reference strain

Kleborate v3.1.2 was used to determine MLST, capsule type, O-locus, virulence genes and score.

AMP, ampicillin; CAZ, ceftazidime; CTX, cefotaxime; MER, meropenem; c*Kp*, classical *K. pneumoniae*; ESBL-c*Kp*, ESBL producing c*Kp*; hv*Kp*, hypervirulent *K. pneumoniae* [virulence score ≥3 or virulence score 2 plus positive string test and/or *rmpA* and/or *rmpA2*, see (Wahl et al., 2024)]; H, human; A, animal; *ybt*, yersiniabactin; *iro*, salmochelin; *iuc*, aerobactin; *clb*, colibactin; *rmpA/rmpA2*, regulators of the mucoid phenotype A and A2; * resistance gene prediction and virulence score according to the latest version of Kleborate v3.1.2 (https://github.com/klebgenomics/Kleborate/wiki/Scores-and-counts); MLST, multilocus sequence type; ^1^, strains used in assays of adhesion, invasion, replication and cytotoxicity; ^2^, strains used in investigations of serum resistance and growth in platelet concentrates.

### DNA and plasmid isolation, PCR and sequencing

Genomic DNA was isolated by various methods for PCR and Sanger sequencing (DNeasy Blood and Tissue Kit, Qiagen, Hilden, Germany; MagAttract HMW DNA Kit, Machery & Nagel, Germany). Presence of beta-lactamase genes (*bla*_TEM-like_, *bla*_SHV-like_, *bla*_CTX-M-Group1&9_, *bla*_NDM-like_, *bla*_OXA-48-like_, *bla*_OXA-1-like_, *bla*_OXA-9-like_) and virulence genes (*rmpA/A2, magA*) was determined using PCR and Sanger sequencing as previously described (Wahl et al., 2024). For whole genome sequencing (WGS), isolates were grown in Brain Heart Infusion (BHI) broth (BD, Heidelberg, Germany). DNA was extracted from overnight cultures using DNeasy Blood and Tissue Kit (Qiagen). DNA was quantified using a Qubit dsDNA HS Assay Kit (Thermo Fisher Scientific). The sequencing libraries were prepared using a Nextera XT DNA Library Prep Kit (Illumina^®^, San Diego, CA, USA). Sequencing was performed according to the manufacturer’s protocol on an Illumina Miseq using v3 chemistry (2 × 300 bp) according to the manufacturer’s protocol. Raw WGS data were processed as described (Wahl et al., 2024). Capsule types and virulence scores were determined using tools like Kleborate (v3.1.2) and Kaptive (Wyres et al., 2020; Lam et al., 2021; Lam et al., 2022). Kleborate is a bioinformatic tool for *K. pneumoniae* allowing species identification, MLST typing and resistance and virulence gene prediction from genomes. Kaptive allows K-locus (capsule) and O-locus (lipopolysaccharide) classification from genome data.

### Cell lines used for cell biological assays

A549 human lung cells (ATCC CCL185, LGC Standards) were cultured in RPMI (Biowest, Nuaillé, France) medium supplemented with 10% FCS. HT29-MTX human colonic epithelial cells were grown in DMEM medium (high glucose, stable glutamine, sodium pyruvate) (Biowest) supplemented with 10% FCS and non-essential amino acids (Biowest). HuTu80 (Cell Line Service) human duodenum cells were kept in DMEM (Biowest) medium (high glucose, stable glutamine, sodium pyruvate) supplemented with 10% FCS. RAW246.7 murine macrophages (ATCC TIB-71™) were cultured in RPMI (sodium pyruvate) (Biowest) supplemented with 10% FCS. 100 U/mL penicillin and 100 μg/mL streptomycin (Biowest) were added to each medium. Cultures were then incubated at 37°C in a humidified atmosphere containing 5% (v/v) CO_2_.

### Adhesion assay

A549 cells and HT29-MTX cells were seeded in 96-well plates (Greiner bio-one Cellstar) at a density of 3×10^3^ cells per well, respectively. The cells were allowed to grow and differentiate for 24 hours at 37°C in a humidified atmosphere containing 5% (v/v) CO_2_. Overnight cultures of the bacterial strains to be analyzed were adjusted in PBS to an OD_600_ of 0.2 (2 × 10^8^ bacterial cells). An inoculum corresponding to a multiplicity of infection (MOI) of 100 (A549) or 25 (HT29-MTX) was prepared in DMEM and used to infect the cells. For the infections, 100 µl of medium was removed from the cells and replaced with 100 µl of the inoculum. After a 5-minute centrifugation step at 500 rpm, the bacteria adhered during a 1 h incubation at 37°C and 5% CO_2_. The culture medium was then removed and non-adherent cells were removed by washing twice with PBS. The cells were lysed with PBS containing 1% Elugent (Merck Millipore, Darmstadt, Germany) and 0,0625% Antifoam B (Sigma-Aldrich, Schnelldorf, Germany) by incubating on a plate rocker at 800 rpm for 20 min at 24°C. 100 µl PBS was added to the lysates and a dilution series (several 10x dilutions) was prepared in PBS, of which 50 µl were plated on MH agar plates. The inoculated agar plates were then incubated overnight at 37°C.

### Invasion assays

A549 and HuTu80 cells were seeded in 96-well plates at a density of 3×10^3^ cells per well, respectively. To determine the invasion rate of *K. pneumoniae* isolates, the cell lines were infected in microtiter plates as described above using a MOI of 100 (A549) or 50 (HuTu80). Cells were washed with PBS after infection and incubated for 1 h in cell culture medium with 100 µg/ml amikacin at 37°C and 5% CO_2_ to kill extracellular bacteria. After 1 h, the extracellular bacteria were removed by washing with PBS and the cells were lysed, diluted (several 10x dilutions) and plated as described above.

### Phagocytosis and replication assays

RAW264.7 cells (ATCC TIB-71™) were seeded in 96-well plates at a density of 3x10^4^ cells per well. To investigate the phagocytosis rate and intracellular replication of *K. pneumoniae*, cells in two microtiter plates were infected in parallel using the desired MOI of 5. The phagocytosis assay was conducted as described for invasion assays. In the second microtiter plate for the intracellular replication assay, the media was replaced with new RPMI with 10 µg/ml amikacin and incubated for 24 h. The cells were then lysed as described above and dilution series (several 10x dilutions) were plated to determine the replication rate using the invasion assay as a reference.

### Cytotoxicity assays

To investigate a cytotoxic effect on intestinal cells, HuTU80 cells were infected with *K. pneumoniae* isolates as described above. Deviating from this, after killing and removal of extracellular bacteria, live/dead staining of the eukaryotic cells was performed using the LIVE/DEAD™ Viability/Cytotoxicity Kit (Thermo Fischer Inc) according to the manufacturer’s protocol.

### Growth behavior in platelet concentrates

Ten *K. pneumoniae* strains (see **Table 1**) were examined with regard to their growth behavior in platelet concentrates. For this purpose, the *K. pneumoniae* strains were grown overnight in LB medium at 37°C with shaking. On the following day, subcultures were inoculated in LB medium with an OD_600_ of 0.1 and incubated up to an OD_600_ of 0.3 at 37°C and 120 rpm. A dilution series of up to 1:100,000 in 0.85% NaCl solution was then prepared from the bacterial suspensions, which corresponds to a cell count of approximately 10 CFU (colony forming unit)/ml. To determine the inoculum, the 10^-5^ dilution, with which the platelet concentrates were later spiked, was plated in triplicates on LB agar plates using a spiral plater (Eddy Jet, Neutec Group, Farmingdale, NY) and incubated overnight at 37°C. Buffy-coat derived pooled platelet concentrates (German Red Cross, Frankfurt, Germany) were pooled in a sterile beaker. As a sterile control, 10 ml of the concentrates were transferred to blood culture bottles and incubated for seven days in the BacT/ALERT system (bioMeriéux, Nürtingen, Germany). Furthermore, the platelet concentrates were transferred to 25 ml blood bags, spiked with 10 CFU of the *K. pneumoniae* isolates to be analyzed and incubated for 43 h at 22.5°C with shaking. After the first 10 h, samples were taken every 3 h, diluted and plated in triplicates with a spiral platter on LB agar plates and incubated overnight at 37°C. The last sample was taken after a total of 43 h. The bacterial colonies were counted automatically using the Sphere Flash apparatus (Neutec Group, Farmingdale, NY).

### Serum resistance assays

In this study, ten *K. pneumoniae* strains (see **Table 1**) were analyzed with regard to their resistance to human serum. For this purpose, the *K. pneumoniae* strains were cultivated overnight at 37°C in LB medium with shaking. On the following day, subcultures were inoculated in LB medium with an OD_600_ of 0.1 and incubated up to an OD_600_ of 0.3 at 37°C and 120 rpm. From the bacterial cultures, 2 ml were pelleted for 5 min at 10000 rpm. The cell pellet was washed with 0.85% NaCl solution, pelleted again for 5 min at 10,000 rpm, and the cell pellet was taken up in 1 ml of 0.85% NaCl solution, which corresponds to a cell count of approximately 2×10^8^ CFU/ml. A dilution series up to 1:100,000 was then prepared from the bacterial suspensions in PAS-II (115.5 mM/l NaCl; 10 mM/l Na-Citrate; 30 mM/l Na-Acetate), corresponding to a cell count of approximately 2×10^3^ CFU/ml. To determine the inoculum, the 10^-5^ dilution, with which the human serum was inoculated, was plated in triplicates on LB medium using a spiral platter and incubated overnight at 37°C. Furthermore, 500 µl of the 10^-5^ dilution was mixed with 500 µl of 50% human serum (Merck, Darmstadt, Germany), which corresponds to a cell count of 10^3^ CFU/ml and a serum concentration of 25% (as in platelet concentrates). The preparations were incubated in a thermoshaker at 37°C and 400 rpm (Eppendorf Thermomixer^®^ C). After 1 h and 4 h, 100 µl of the cultures were plated in triplicates with using a spiral plater on LB agar plates and incubated overnight at 37°C. The next day, the bacterial colonies were automatically counted using the Sphere Flash apparatus.

### Light microscopy

For microscopy of the cell morphology of the different *K. pneumoniae* pathotypes (c*Kp*, ESBL-c*Kp*, hv*Kp*), various isolates were cultivated at 37°C until the stationary growth phase (isolates 16-0382, 16-0383, 17-0683, 17-0684, 17-0609, 18-0005; see **Table 1**). One ml of the bacterial culture was centrifuged at 14,000 rpm for 5 min. The cell pellet was resuspended in 1 ml crystal violet solution (50 µl crystal violet + 950 µl PBS) and incubated for 1 min. The culture was then centrifuged again and the cell pellet was washed in PBS. A 60% ink solution (in PBS) was added to the bacterial suspension at a ratio of 1:2. 0.2 µl of the ink stained suspension was added to a slide coated with 1.5% agarose (in PBS) and covered with a coverslip. The phase contrast of the Nikon Eclipse Ti microscope was used to analyze the cell morphology of the bacterial cells under a 100× oil immersion objective and the images were recorded using a Nikon DS-MBWc CCD camera. To determine the cell lengths, 100 bacterial cells per strain were measured in triplicates using the MicrobeJ program. MicrobeJ is a software plugin tool developed for ImageJ to detect and count bacterial cells (https://www.microbej.com/; last access 08.01.2025).

### Statistical analysis

Statistical analysis was conducted using GraphPad Prism or R software. Unless otherwise specified, all analyses were performed based on three independent experiments. The results were presented as mean ± standard deviation (S.D.). Detailed information regarding the specific statistical tests employed for each analysis can be found in the corresponding figure legends.

## Results

### Cell morphological differences of *K. pneumoniae* pathotypes

**Figure 1:**
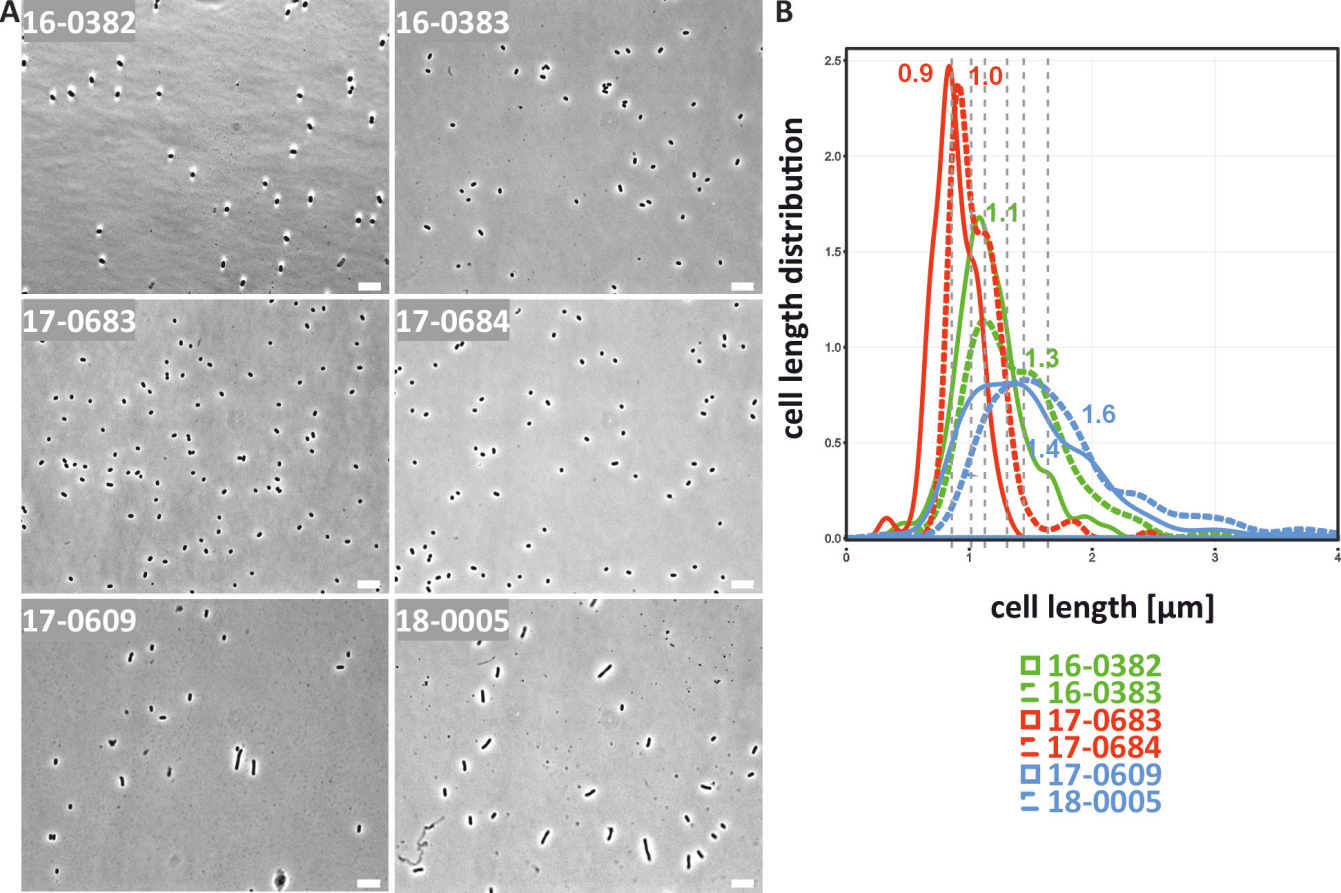
Cell morphological differences of c*Kp*, ESBL-c*Kp* and hv*Kp* isolates under normoxia. **(A)** Light microscopic images of c*Kp* (17-0683 and 17-0684), ESBL-c*Kp* (16 0382 and 16-0383) and hv*Kp* isolates (17-0609 and 18-0005) showed a difference in capsule thickness and cell length. **(B)** The distribution of cell lengths of the analyzed *K*. *pneumoniae* isolates is shown. Using MicrobeJ, triplicates of each strain with 100 cells each were measured, analyzed with R and visualized.

The six clinical *K. pneumoniae* isolates 16-0382, 16-0383, 17-0683, 17-0684, 17-0609, 18-0005 (see **Table 1**) were analyzed for possible cell morphological differences using ink staining and light microscopy. Based on the microscopic images, the thick capsular layer known for hv*Kp* was observed and appears as a clear white halo around the bacterial cells (**Figure 1A**). In addition, clear differences in the cell length of the hv*Kp* in comparison to the c*Kp* and the ESBL-c*Kp* isolates were observed. As can be seen in **Figure 1**, the cells of the hv*Kp* isolates are longer and have a filamentous shape, while the c*Kp* and ESBL-c*Kp* isolates have a rod shape typical of bacteria of the *Enterobacterales* family. In order to quantify this result, the cell lengths distribution was analyzed as visualized in microscopic images. This revealed that the median cell length of hv*Kp* isolates is longer than that of c*Kp* and ESBL-c*Kp* isolates. In addition, the hv*Kp* isolate 18-0005 had longer cells than hv*Kp* isolate 17-609.

### Cell morphological changes of *K. pneumoniae* pathotypes under hypoxia

**Figure 2:**
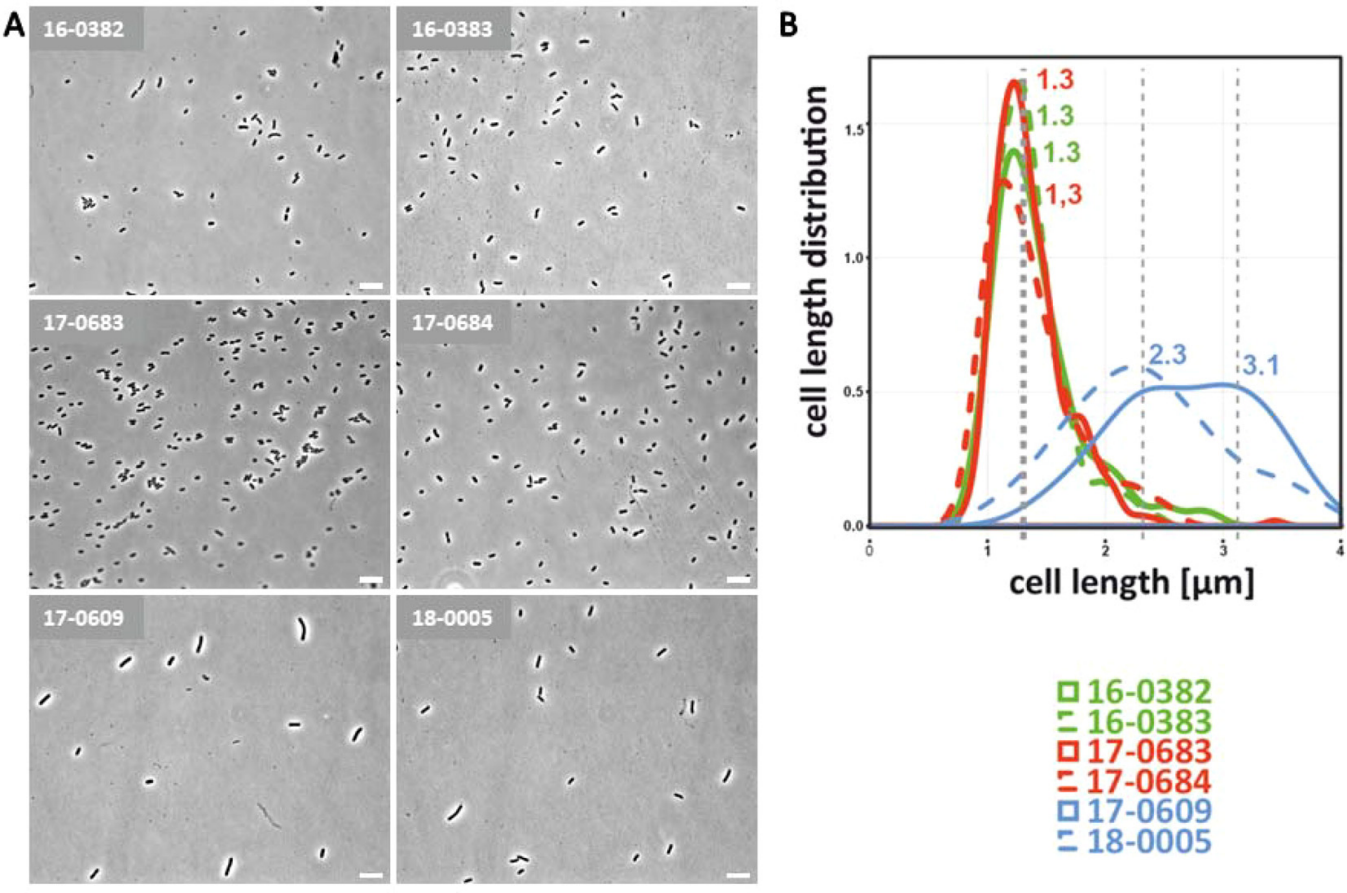
Cell morphological differences of c*Kp*, ESBL-c*Kp* and hv*Kp* isolates under hypoxia. **(A)** Light microscopic images of c*Kp* (17-0683 and 17-0684), ESBL-c*Kp* (16 0382 and 16-0383) and hv*Kp* isolates (17-0609 and 18-0005) showed a difference in capsule thickness and cell length. **(B)** Distribution of cell lengths of analyzed *K*. *pneumoniae* isolates. Using MicrobeJ, triplicates of each strain with 100 cells each were measured, analyzed with R and visualized.

*K. pneumoniae* is a facultative anaerobic bacterium that grows optimally in the presence of oxygen, but can also survive in low-oxygen environments such as the intestine. For *E. coli*, it has already been shown that they react to oxygen reduction by filamentation of the bacterial cells and that this has an influence on the pathogenicity (Murashko and Lin-Chao, 2017). It should therefore be investigated whether cell morphological changes in *K. pneumoniae* can also be observed under oxygen reduction. For this purpose, the isolates were cultivated overnight at an oxygen concentration of 0.5 vol% and analyzed microscopically. As shown in **Figure 2**, an elongation of the bacterial cells was observed in light microscopic images and cell length measurements for representatives isolates of all three pathotypes. As previously shown under normoxia, the bacterial cells of the hv*Kp* isolates were longer than those of the c*Kp* and ESBL-c*Kp* isolates. The results show an altered cell length of *K. pneumoniae* depending on oxygen. Due to the similarity to *E. coli* in the O_2_-dependent length distribution, we also suspect an influence of O_2_ on the pathogenic potential of *K. pneumoniae*.

### *In vitro* adhesion experiments

**Figure 3:**
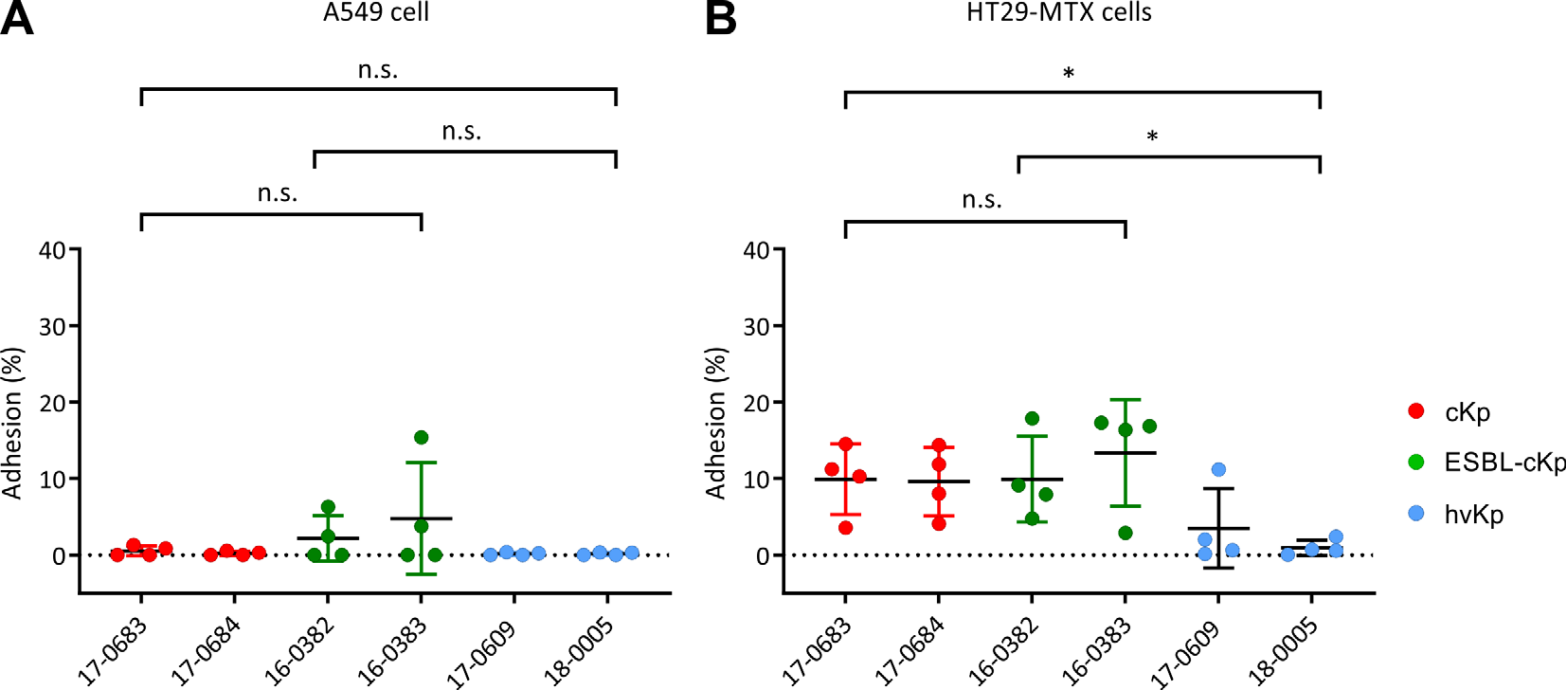
Quantification of the adherence of *K. pneumoniae* pathotypes to A549 and HT29-MTX cells. The adherence of c*Kp* (17-0683 and 17-0684), ESBL-c*Kp* (16-0382 and 16-0383) and hv*Kp* isolates (17 0609 and 18-0005) to **(A)** A549 lung cells and **(B)** HT29-MTX intestinal cells is shown as a percentage of the inoculum. The data represent the results from four biologically independent triplicates. ANOVA was applied to the data to calculate significance between groups. Statistical significance is indicated as follows: n.s., not significant, *P ≤ 0.05.

Studies have shown that elongated bacterial cells can adhere better to epithelial cells *in vitro* due to their enlarged cell surface (Yang et al., 2016). As described above, a filamentous form was observed for the bacterial cells of hv*Kp* isolates 17-0609 and 18-0005. In the following, it was investigated whether a difference in adhesion can be observed between the different *K. pneumoniae* pathotypes. Because *K. pneumoniae* colonizes the respiratory and intestinal tracts, the lung cell line A549 and the intestinal cell line HT29-MTX were selected as *in vitro* cell models. **Figure 3** shows the adhesion rates as a percentage of the inoculum. The *K. pneumoniae* isolates analyzed adhered to a lesser extend to A549 lung cells *in vitro* than to HT29-MTX intestinal cells. The adhesion rate was below 5% (**Figure 3A**). Furthermore, no differences in adhesion capacity were observed between the different *K. pneumoniae* pathotypes on A549 lung cells. Although the standard deviation was large due to fluctuating adhesion rates in the individual experiments, a trend could be recognized from the data showing a better adhesion of c*Kp* cells (independent from the ESBL status) than hv*Kp* cells (**Figure 3B**). The analyzed c*Kp* and ESBL-c*Kp* isolates showed a higher adhesion rate (about 10% of the inoculum) to intestinal cells than to lung cells in the *in vitro* cell culture model. In contrast, the adhesion rate of hv*Kp* isolates was significantly lower, the adhesion rate here was also < 5%. Thus, the results obtained in the infection experiments do not indicate a positive influence of cell morphology on the adhesion capacity.

### *In vitro* cellular invasion assays

**Figure 4:**
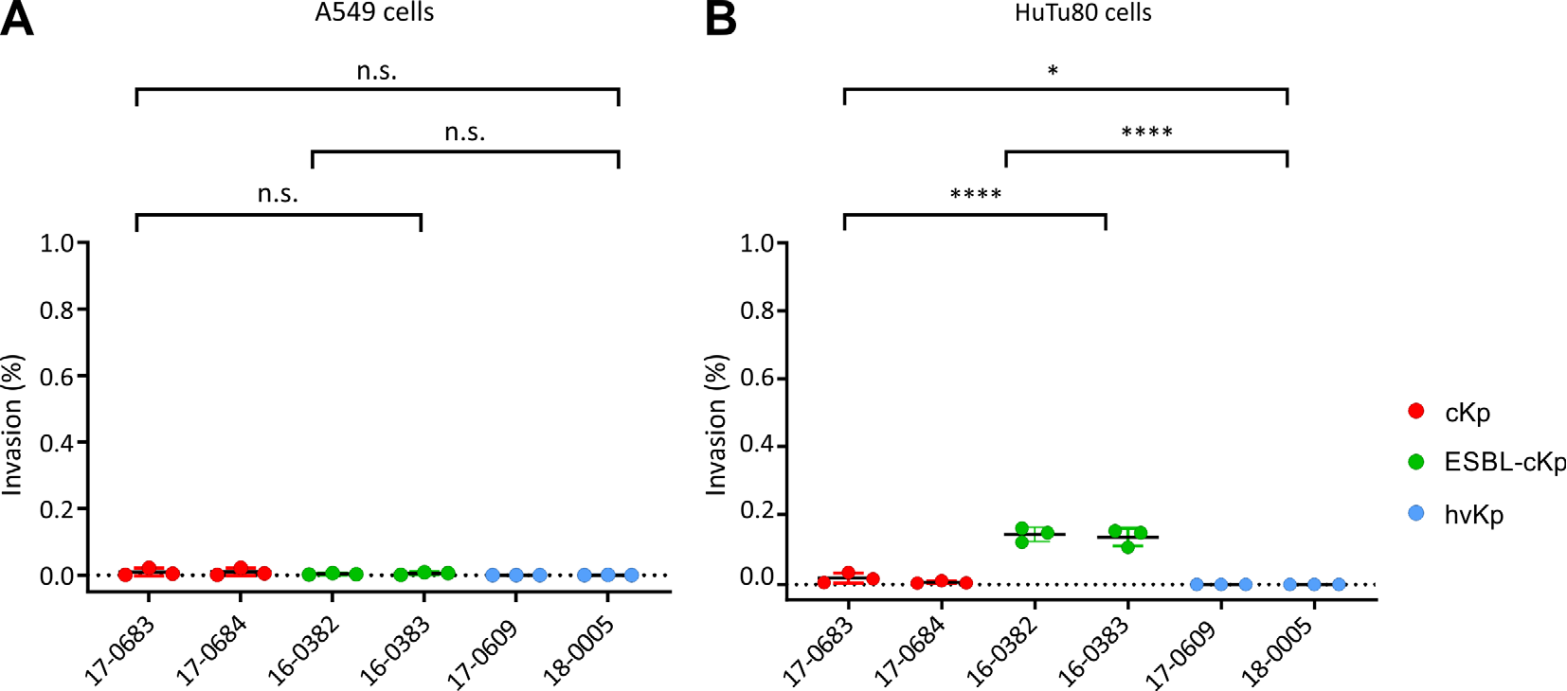
Quantification of the invasion of *K. pneumoniae* pathotypes in A549 and HuTu80 cells. The invasion of c*Kp* (17-0683 and 17-0684), ESBL-c*Kp* (16 0382 and 16-0383) and hv*Kp* isolates (17-0609 and 18-0005) in **(A)** A549 lung cells and **(B)** HuTu80 intestinal cells in relation to the inoculum. The data represent the results from three biologically independent triplicates. ANOVA was applied to the data to calculate significance between groups. Statistical significance is indicated as follows: n.s., not significant, *P ≤ 0.05, ****P ≤ 0.0001.

We investigated whether a difference in cellular invasion can be observed between the included representative isolates of the three *K. pneumoniae* pathotypes. To determine the cellular invasion, the lung cell line A549 and the intestinal cell line HuTu80 were selected as models. **Figure 4** shows the invasion rates as a percentage of the inoculum. The *K. pneumoniae* isolates analyzed showed very low invasion *in vitro* (invasion rate <1%) in A549 lung cells (**Figure 4A**) and HuTu80 intestinal cells (**Figure 4B**). As previously shown in the adhesion model, no significant differences were found between the different *K. pneumoniae* pathotypes with regard to invasion in A549 lung cells. However, differences were observed in the invasion rates determined in the cell culture model with HuTu80 intestinal cells. Here, the two ESBL-c*Kp* isolates showed a significantly higher invasion rate than the two c*Kp* and the two hv*Kp* isolates. The results obtained with the infection models used did not show increased cellular invasion of the hv*Kp* isolates compared to the c*Kp* isolates analyzed.

### *In vitro* phagocytosis experiments

**Figure 5:**
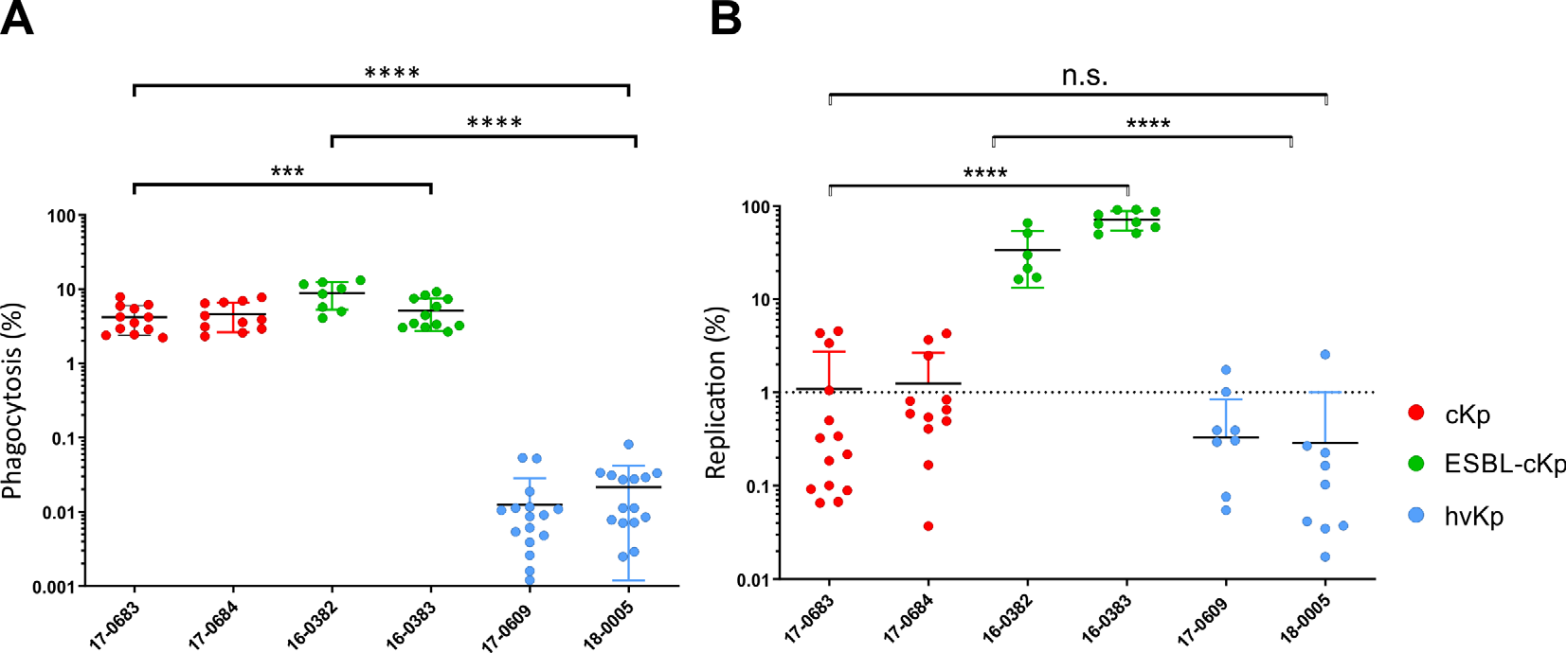
Quantification of phagocytosis resistance and intracellular replication of different *K*. *pneumoniae* pathotypes. **(A)** Phagocytosis rate of c*Kp* (17-0683 and 17-0684), ESBL-c*Kp* (16 0382 and 16-0383) and hv*Kp* isolates (17-0609 and 18-0005) by murine RAW 264.7 macrophages (60,000 cells/cavity) after one hour of infection. **(B)** Replication rate of c*Kp* (17-0683 and 17-0684), ESBL-c*Kp* (16-0382 and 16-0383) and hv*Kp* (17-0609 and 18-0005) isolates in murine RAW 264.7 macrophages (60,000 cells/cavity) after 24 hours of infection. ANOVA was applied to the data to calculate significance between groups. Statistical significance is indicated as follows: n.s., not significant, ***P ≤ 0.01 and ****P ≤ 0.0001.

Using an *in vitro* infection model with murine RAW 264.7 macrophages, the interaction of the *K. pneumoniae* isolates with phagocytes was determined. The aim was to characterize possible differences in phagocytosis resistance between the representative isolates of the different *K. pneumoniae* pathotypes. To this end, the phagocytosis rate of the six different *K. pneumoniae* strains was first compared. After a 1 h of infection, the number of intracellular bacteria was determined. The phagocytosis rate was calculated in relation to the inoculum. **Figure 5A** shows the phagocytosis rate of the six *K. pneumoniae* strains from three independent experiments. The hv*Kp* isolates 17-0609 and 18-0005 showed a significantly lower phagocytosis rate *in vitro* compared to c*Kp* and ESBL-c*Kp* isolates (16-0382 and 16-0383).

To clarify whether the investigated *K. pneumoniae* strains survive and/or replicate intracellularly in RAW 264.7 macrophages, the number of intracellular bacterial cells was determined 24 h after infection and the replication rate was calculated in relation to the phagocytosis rate. The hv*Kp* isolates 17-0609 and 18-0005 were killed within the macrophages as indicated by a replication rate of less than one (**Figure 5B**). A replication rate of about one for c*Kp* isolates 18-0683 and 18-0684 indicated intracellular survival without replication in mouse macrophages. In contrast, both ESBL-c*Kp* isolates showed *in vitro* replication rates of about 90-fold. They can persist and replicate intracellularly in macrophages.

### Comparisons of intracellular replication of *K. pneumoniae* pathotypes under normoxia and hypoxia

**Figure 6:**
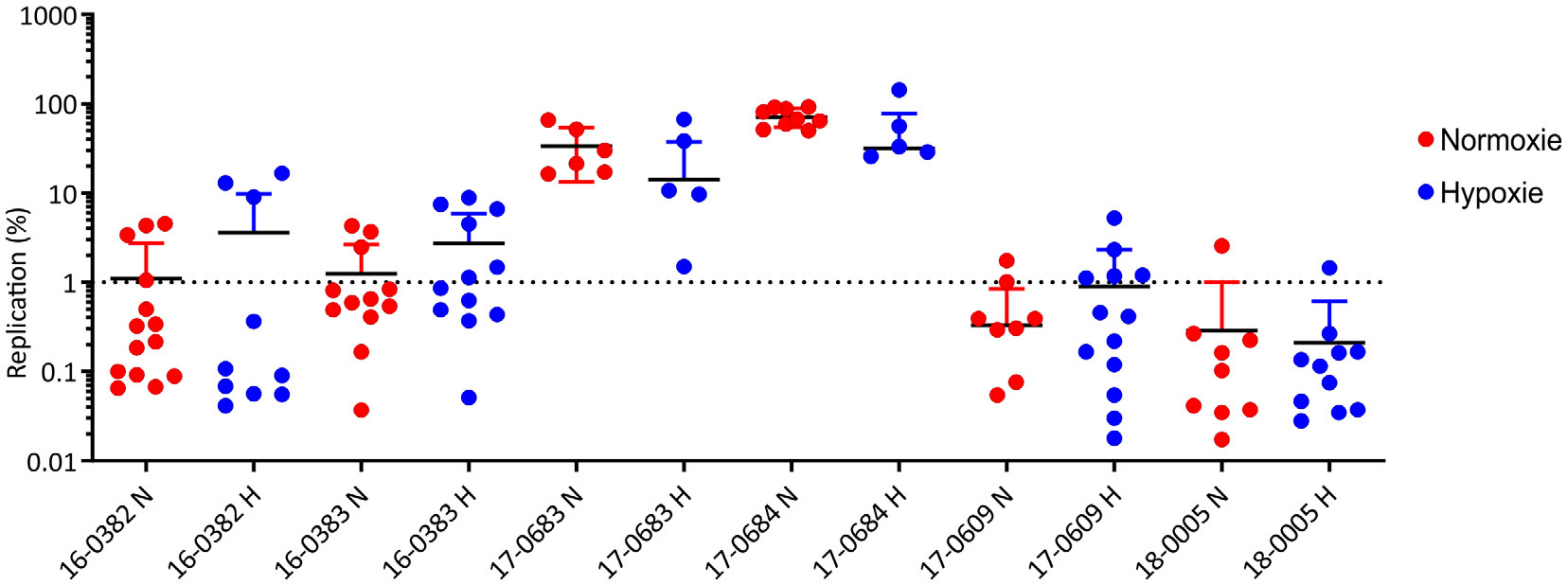
Comparison of the replication of *K. pneumoniae* isolates representing different pathotypes in RAW 264.7 mouse macrophages under normoxia (N, red) and hypoxia (H, blue). The replication rates of c*Kp* (17-0683 and 17-0684), ESBL-c*Kp* (16-0382 and 16-0383) and hv*Kp* isolates (17-0609 and 18-0005) in murine RAW 264.7 macrophages (60,000 cells/cavity) under normoxia (N; O_2_ = 20.9%; red) and hypoxia (H; O_2_ = 0.5%; blue) are shown.

For some enteropathogenic bacteria such as *Salmonella*, it has already been shown that a reduction in oxygen is associated with increased virulence of the pathogen (Marteyn et al., 2011). After filamentation of the *K. pneumoniae* cells under oxygen reduction has been observed (**Figure 2**), the aim was to investigate whether reduced oxygen availability also has an influence on the interaction with host cells. For this purpose, mouse macrophages were infected in parallel in two cell culture plates. One plate was incubated as usual under normoxia (O_2_ = 20.9%) and the second plate under hypoxia (O_2_ = 0.5%) for 24 hours. **Figure 6** shows the replication rates of the analyzed six *K. pneumoniae* isolates. No significant difference was found in the replication rates under hypoxia. Again, only the ESBL-c*Kp* isolates were able to replicate, while c*Kp* isolates persisted and hv*Kp* isolates died. No difference in intracellular replication was observed comparing hypoxic and normoxic culture conditions of the individual strains.

### Cytotoxic effects of clinical *K. pneumoniae* pathotypes

**Figure 7:**
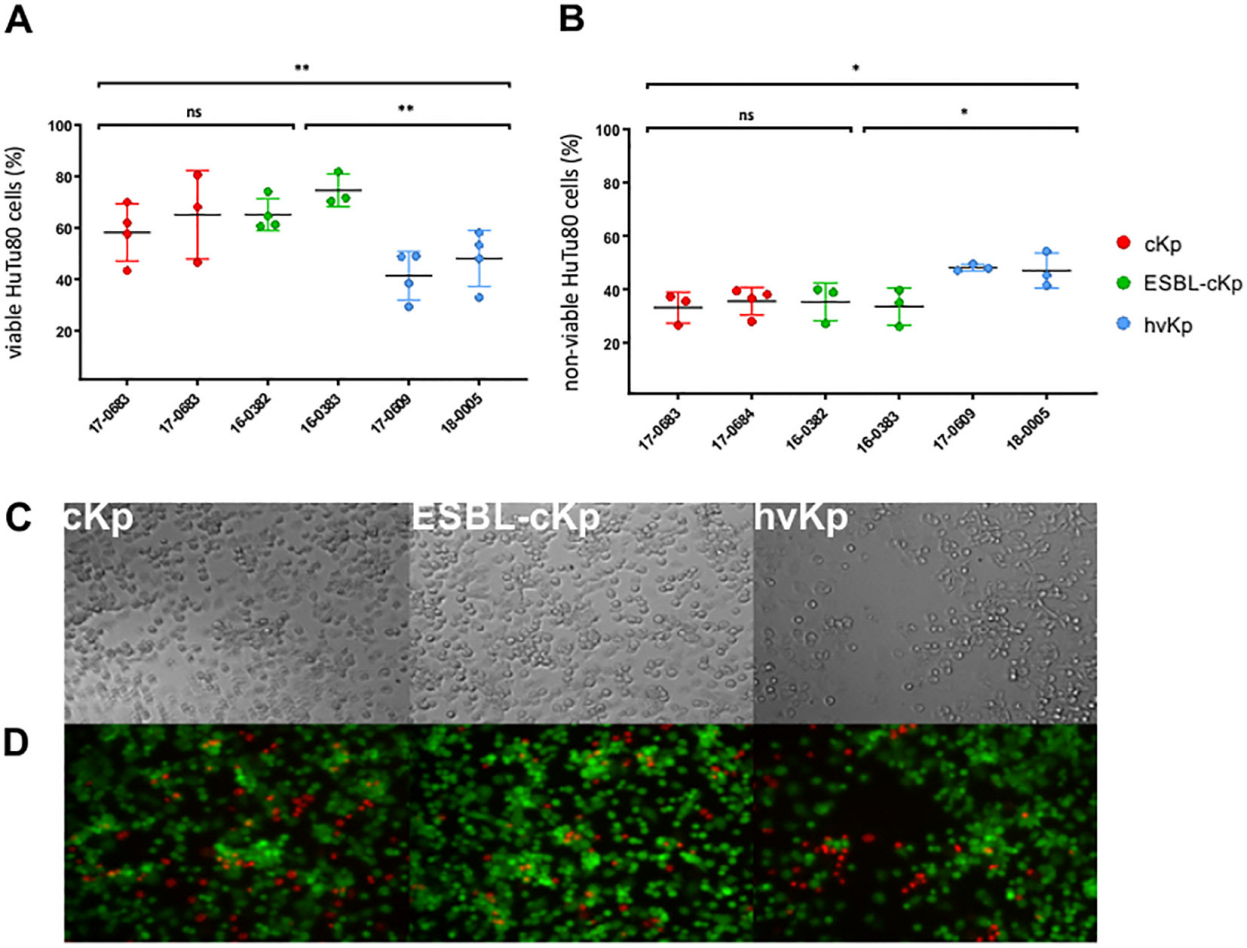
Comparison of the cytotoxic effect of *K*. *pneumoniae* pathotypes on HuTu80 intestinal cells, showing the percentage of viable **(A)** and non-viable **(B)** HuTu80 intestinal cells after infection with c*Kp* (17-0683 and 17-0684), ESBL-c*Kp* (16 0382 and 16-0383) and hv*Kp* isolates (17-0609 and 18-0005). The data represent the results of triplicates from three independent experiments. ANOVA was applied to the data to calculate significance between groups. Statistical significance is indicated as follows: *P ≤ 0.05 and **P ≤ 0.01. Transmitted light microscopy **(C)** and fluorescence microscopy **(D)** images of viable (green) and non-viable (red) HuTu80 intestinal cells after infection with *K*. *pneumoniae*. n.s., not significant.

As described above, no cellular invasion of intestinal cells was observed for hv*Kp* isolates. However, studies by Cano et al. had shown a cytotoxic effect of *K. pneumoniae* on epithelial cells (Cano et al., 2009). Therefore, we investigated whether the six *K. pneumoniae* isolates 16-0382, 16-0383, 17-0683, 17-0684, 17-0609 and 18-0005 have a cytotoxic effect on intestinal cells. For this purpose, HuTu80 intestinal cells were infected with the *K. pneumoniae* isolates as previously described. Live/dead staining with calcein AM (viable) and ethidium bromide (non-viable) was used to discriminate between viable and non-viable HuTu80 cells, which were analyzed both photometrically and microscopically. **Figure 7** shows the percentages of viable and non-viable HuTu80 cells. All investigated c*Kp*, ESBL-c*Kp* and hv*Kp* isolates showed a cytotoxic effect on intestinal cells *in vitro*. The viability of HuTu80 cells after infection was below 80% for isolates of all pathotypes and the percentage of non-viable cells was above 20%. Hv*Kp* isolates showed a stronger cytotoxic effect *in vitro* than c*Kp* and ESBL-c*Kp* isolates. Microscopic images of cells infected with hv*Kp* isolates showed the loss of integrity of the HuTu80 monolayer, whereas a confluent cell lawn persisted after infections with c*Kp* and ESBL-c*Kp* isolates (**Figure 7C**).

### Growth of *K. pneumoniae* isolates in platelet concentrates

**Figure 8:**
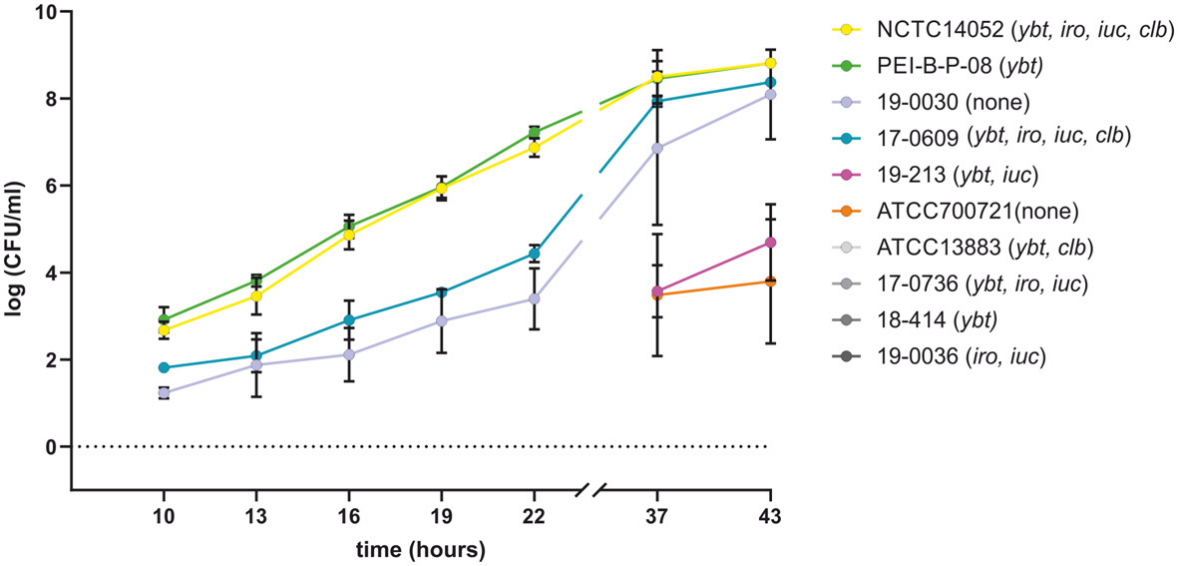
Comparison of the growth behavior of *K. pneumoniae* isolates in platelet concentrates. The growth curves of *K. pneumoniae* isolates ATCC 13883, ATCC 700721, NCTC 14052, PEI-B-P-08, 17-0609, 17-0736, 18-0030, 18-0414, 19-0036 and 19-0213 at 22.5°C in platelet concentrates are shown. No growth could be detected for the four isolates labelled with grey colors. The data represent the results of triplicates from three independent experiments.

The growth behavior of 10 K*. pneumoniae* isolates, which harbor different siderophore genes (**Table 1**), was compared with that of the *K. pneumoniae* reference strain PEI-B-P-08 [143] in platelet concentrates. For this purpose, a 50 ml platelet concentrate was spiked with 10 CFU of one of the *K. pneumoniae* isolates to be tested and incubated under conditions specified by haemotherapy guidelines (Spindler-Raffel et al., 2017; Prax et al., 2019). **Figure 8** shows the growth kinetics of the analyzed *K. pneumoniae* isolates over a period of 43 hours. For reference strain PEI-B-P-08 (c*Kp*), hv*Kp* reference strain NCTC 14052 and test strains 18-0030 (c*Kp*) and 17-0609 (hv*Kp*), an increase in bacterial count was observed after 10 h, with the bacterial count of isolates NCTC 14052 and PEI-B-P-08 being one order of magnitude higher than that of isolates 18-0030 and 17-0609. Furthermore, the growth of isolates NCTC 14052 and PEI-B-P-08 was exponential, while the growth of isolates 18-0030 and 17-0609 showed a linear increase. After 37-43 h, the growth of isolates NCTC 14052, PEI-B-P-08, 18-0030 and 17-0609 was in the stationary phase with bacterial counts of about 10^7^. The isolates 19-0213 (hv*Kp*) and ATCC 700721 (c*Kp*) exhibited a long lag phase of over 22 h. Only after 38 h a significant increase in bacterial counts was detected for these isolates. Four further isolates analyzed (ATCC13883 (c*Kp*), 17-0736 (hv*Kp*), 18-0414 (ESBL-c*Kp*) and 19-0036 (hv*Kp*) were unable to grow in platelet concentrates. This growth behavior could be due to different serum resistances (Doorduijn et al., 2016).

### Serum resistance of *K. pneumoniae* isolates

**Figure 9:**
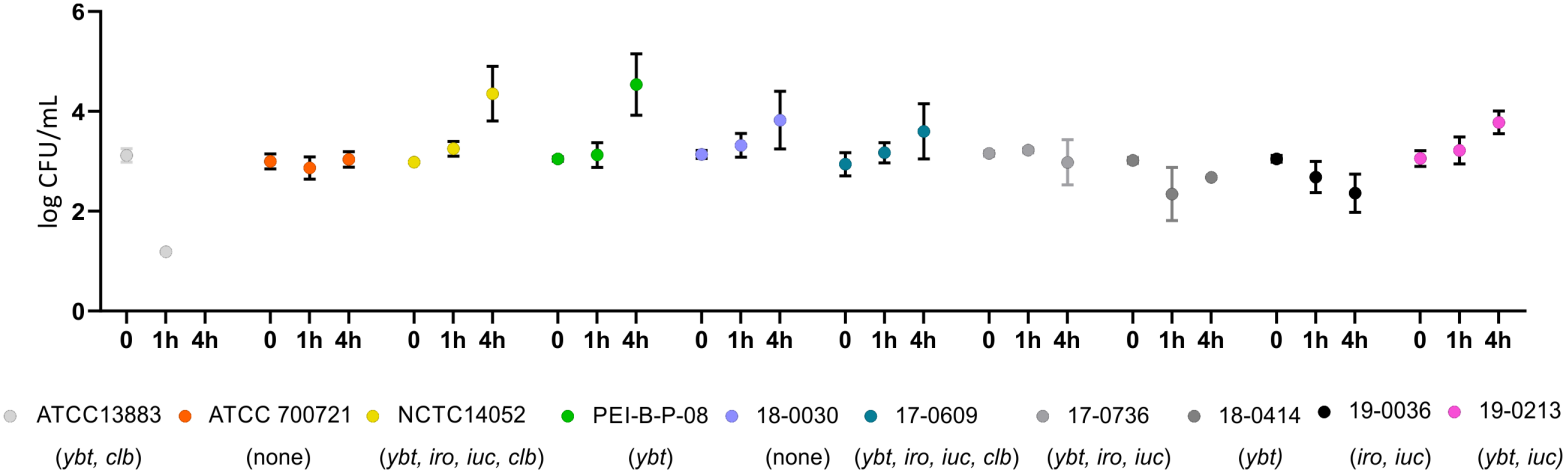
Comparison of the serum resistance of ten *K. pneumoniae* isolates. Serum resistance of *K. pneumoniae* isolates ATCC 13883, ATCC 700721, NCTC 14052, PEI-B-P-08, 17-0609, 17-0736, 18-0030, 18-0414, 19-0036 and 19-0213 at 37°C in 25% human serum. No growth could be detected for isolates labelled in different grey colors. The data represent the results of triplicates from three independent experiments.

In order to investigate possible serum resistance of the ten *K. pneumoniae* isolates, human serum was inoculated with 10^3^ CFU of the isolates to be analyzed. **Figure 9** shows the results of the bacterial counts determined after 1 h and 4 h. The isolates PEI-B-P-08 (c*Kp*), NCTC 14052 (hv*Kp*), 17-0609 (hv*Kp*) 18-0030 (c*Kp*), 19-0213 (hv*Kp*) and ATCC 700721 (c*Kp*), were resistant to human serum. While the bacterial counts of isolates ATCC 700721 remained stable, increased bacterial counts were observed for isolates NCTC 14052, PEI-B-P-08, 18-0030, 17-0609, and 19-0213. The isolates ATCC 13883 (c*Kp*), 17-0736 (hv*Kp*), 18-0414 (ESBL-c*Kp*) and 19-0036 (hv*Kp*) are not resistant to human serum, here bacterial counts decreased over 4 h.

## Discussion

Previous studies have focused on genome-wide association studies of globally distributed *K. pneumoniae* lineages (“high-risk clones”) and epidemiological studies to identify risk factors that favor nosocomial *K. pneumoniae* infections (Cano et al., 2009; Meatherall et al., 2009; Martin and Bachman, 2018; Cienfuegos-Gallet et al., 2019). However, in the face of increasing antibiotic resistance and the global spread of hypervirulent strains, a better understanding of the pathogenesis of *K. pneumoniae* is essential for the development of preventive measures and more targeted treatments including “anti-virulence strategies”. Therefore, the present study investigated properties of different *K. pneumoniae* pathotypes in order to be able to make statements about host adaptations that contribute to the successful spread of this bacterium in the different habitats.

First, differences in the cell morphology of hv*Kp* isolates compared to c*Kp* and ESBL-c*Kp* isolates were determined and documented by means of light microscopy (**Figures 1**, **2**). The analyzed hv*Kp* isolates showed a filamentous phenotype. In the literature, filamentation of bacterial cells is often described as the result of impaired cell division (Klein et al., 2015; Cayron et al., 2023). However, some bacteria are able to react to stress conditions such as oxidative stress, nutrient deficiency or the defense mechanisms of the host immune system by a temporary change in cell morphology (Justice et al., 2008). For *E. coli*, it has been observed that the enlargement of the cell surface associated with a filamentous phenotype has a positive effect on adhesion to surfaces and protects the bacteria from phagocytosis by immune cells (Klein et al., 2015). In our study, cell filamentation of hv*Kp* was much more pronounced when compared to c*Kp* and ESBL-c*Kp*, but cell lengths’ variation among individual hv*Kp* isolates was also remarkable suggesting a potential greater polymorphic population than among c*Kp* and ESBL-c*Kp.* However, adhesion of hv*Kp* to two different cell lines was weaker compared to c*Kp* (**Figures 1**–**3**). Thus, the positive effect of cell filamentation might be compensated by the protective effect of the larger capsule (**Figure 3**). Under hypoxia, an increase in cell length was also observed for c*Kp* and ESBL-c*Kp* isolates, but to a much lesser extent than for hv*Kp* isolates. It is known that filamentous bacteria have an advantage in adherence to epithelial cells. As hypoxia conditions prevail in the gut, reduced oxygen levels could be a reason for *K. pneumoniae* to alter cell morphology in order to adhere better to the intestinal epithelium.

The cell culture model was used to investigate whether representative isolates of the three *K. pneumoniae* pathotypes show differences in the pathogen-host interaction. Since respiratory and gastrointestinal colonization of the bacterium is considered to be the main reservoir for *K. pneumoniae* infections, the cell infection experiments were carried out with the lung cell line A549 and the intestinal cell lines HT29-MTX and HuTu80 (Sun et al., 2019). In previous cell infection experiments, the adhesion and cellular invasion of encapsulated and non-encapsulated *K. pneumoniae* isolates were compared (Sahly et al., 2000; Cortes et al., 2002; Struve and Krogfelt, 2003; Opoku-Temeng et al., 2019). A direct correlation between the adhesion to and invasion of lung epithelial cells and the ability of *K. pneumoniae* isolates to express capsular polysaccharide has been observed (Fumagalli et al., 1997; Sahly et al., 2000; Struve and Krogfelt, 2003; de Astorza et al., 2004). No differences of the included representative strains representing the three *K. pneumoniae* pathotypes c*Kp*, ESBL-c*Kp* and hv*Kp* in adherence to and invasion of A549 lung cells were found (**Figures 3A**, **4A**). In general, adhesion/invasion rates are lower with the lung cell lines compared to intestinal cells. These results suggest that adhesion and invasion of lung cells is not a classical pathophysiological mechanism of *K. pneumoniae*. One possible way in which the bacterium is able to overcome the cell barrier in the respiratory tract is by disrupting the cell integrity of the epithelium through injury in the respiratory tract or, as described by Cano et al., via a cytotoxic effect on A549 lung cells by encapsulated *K. pneumoniae* isolates (Cano et al., 2009).

In the intestinal cell model, however, differences in adherence and invasion between the isolates representing the three different *K. pneumoniae* pathotypes were observed (**Figures 3B**, **4B**). While the c*Kp* and ESBL-c*Kp* isolates are equally capable of adhering to intestinal cells, no adhesion was observed for the hv*Kp* isolates analyzed. These results are congruent with the observations that a thick capsular layer reduces the adhesion capacity of *K. pneumoniae* (Sahly et al., 2000). Furthermore, the hypothesis that an enlarged cell surface increases adhesion due to a filamentous phenotype was not confirmed under normoxic conditions. Hus et al. were able to show in a cell culture model that *K. pneumoniae* can overcome the barrier function of the intestinal epithelium via a transcellular pathway (Hsu et al., 2015). This could only be confirmed for ESBL-c*Kp* in the present study. The c*Kp* and hv*Kp* isolates showed no cellular invasion *in vitro* in the intestinal cell line used. These results are comparable to previous data by Sahly et al. who observed an increased cellular invasion of ESBL-producing *K. pneumoniae* isolates (Sahly et al., 2008), an effect that might potentially derive from additional features encoded on ESBL-containing plasmids than from the pure presence of the ESBL gene itself.

The clinical pictures of hv*Kp* infections include invasive and necrotizing infections, which suggests a cytoxic effect of hv*Kp* isolates (Evangelista et al., 2018). Our study showed that the investigated hypervirulent isolates had a significantly higher cytotoxic effect on intestinal epithelial cells than c*Kp* or ESBL-c*Kp* isolates. Holes in the monolayer were observed after infection demonstrating the loss of epithelial integrity (**Figure 7C**). It can be assumed that the proportion of non-viable cells was even higher, as the detached (and subsequently washed away) cells could not be detected. This indicates that hv*Kp* do not invade intestinal cells but (**Figure 4**), due to their cytotoxic effect, damage the intestinal epithelium and overcome the intestinal barrier in this way. Recent studies in particular describe virulence factors that support this hypothesis. For example, a genotoxic effect on eukaryotic cells was observed *in vitro* for colibactin-producing *K. pneumoniae* isolates (Lai et al., 2014; Lu et al., 2017). It is assumed that colibactin leads to apoptosis of intestinal cells and thus enables the intestinal translocation of hv*Kp* (Secher et al., 2015; Wyres et al., 2020).

The capsule of *K. pneumoniae* is the most important virulence factor (Huynh et al., 2017; Opoku-Temeng et al., 2019). It protects the bacteria from antibiotics, the complement system of the immune defense and phagocytosis (Kabha et al., 1995). It is known from the literature that encapsulated *K. pneumoniae* are resistant to opsonization *in vivo* and to uptake by phagocytes (Domenico et al., 1994; Alvarez et al., 2000). The studies also compared encapsulated *K. pneumoniae* isolates and capsule mutants with each other. However, only the general role of the capsule on host interaction was investigated. No conclusions can be drawn about differences between the *K. pneumoniae* pathotypes. The present study investigated the extent to which the abilities of phagocytosis resistance, intracellular persistence and replication differ between the included isolates representing the various *K. pneumoniae* pathotypes. Using a mouse macrophage infection model, significant differences were observed, both c*Kp* and ESBL-c*Kp* were equally phagocytosed by the immune cells, while the hv*Kp* isolates were not (**Figure 5**).

Our results also showed no intracellular persistence or replication for hv*Kp* isolates (**Figure 6**). There are several possible reasons for this. Firstly, increased capsule synthesis is characteristic of hv*Kp* isolates, which makes them less accessible to macrophages. Furthermore, as described above, filamentation of the hv*Kp* bacterial cells was observed. For some bacteria it is known that they change from the bacillary to the filamentous cell form and are therefore less accessible for phagocytotic cells (Prashar et al., 2013). However, the comparatively low number of phagocytosed hv*Kp* must be considered as a limitation, which led to a higher variability of the individual experiments despite a large number of replicates.

The ability of ESBL-c*Kp* to increase intracellular persistence compared to c*Kp* as described by Cano et al. was confirmed in the present study (Cano et al., 2015) (**Figures 5**, **6**). Interestingly, the ESBL-*Kp* isolates analyzed were also able to replicate in RAW macrophages. While hv*Kp* were able to evade macrophage access, ESBL-c*Kp* have found a strategy to replicate in macrophages similar to classical intracellular pathogens such as *Legionella pneumophila* and *Salmonella enterica* (Price and Vance, 2014).

There is currently a lack of a standardized method for virulence prediction of *K. pneumoniae* and thus a derivation of markers for laboratory diagnostics. To date, virulence analyses have concentrated on genome comparisons and genome-wide association studies (Spadar et al., 2022; Cheung et al., 2023). A conventional assessment of the virulence potential and the differentiation of c*Kp* and hv*Kp* isolates is currently under discussion and is based, among other things, on the detection of specific iron transport systems (Holt et al., 2015; Lam et al., 2018b; Lam et al., 2018a). Molecular epidemiological studies have shown that the prevalence of iron transport systems is higher in hv*Kp* isolates than in c*Kp* isolates (Russo et al., 2015; Russo and Marr, 2019).

In this study, the growth of hv*Kp* isolates with different siderophore systems (**Table 1**) was compared with that of the reference strain PEI-BP-08 in platelet concentrates. The PEI-BP-08 reference isolate (ST48-K62), which only possesses *ybt*, and the hv*Kp* reference strain NCTC 14052 (ST23-K1) harboring all relevant siderophore genes (*ybt, iro, iuc, clb)* and capsule regulator genes (*rmpA/rmpA2*), in addition, were among the fast-growing isolates. c*Kp* isolate 18-0030 (ST310-K1) and hv*Kp* isolate 17-0609 (ST3895-K2) were also able to proliferate in platelet concentrates, but their proliferation was delayed. Interestingly, for the hv*Kp* isolate 19-0213 (ST23-KL107) and ESBL-c*Kp* isolate ATCC 700721 (ST38-K15), proliferation was only detected after an incubation period of 37 hours. Bacteria with such a long lag phase represent a major challenge in transfusion medicine, as such bacterial contamination is difficult to detect and harbors the risk of transfusion-related bacterial infections. Despite the presence of siderophores, no growth could be observed for four of the analyzed isolates of various classifications and with presence of several siderophore genes.

In contrast to the present knowledge and working hypotheses (Holt et al., 2015; Russo and Marr, 2019), our study results indicate that siderophores are not essential factors for the proliferation of *K. pneumoniae* in blood components. It was shown that virulence prediction by the presence or absence of siderophores alone is not necessarily associated with the ability to proliferate in blood components, which calls into question the reliability of the proposed classification of hv*Kp* isolates based on the presence of specific iron transport systems only (Holt et al., 2015; Lam et al., 2021). Instead, the results of the present work provide evidence that the capsule plays the decisive role in the pathogenesis of *K. pneumoniae*. Current studies are also increasingly focusing on the investigation of the capsule of *K. pneumoniae* and its regulation and expression as well as synthesis mechanisms (Chu et al., 2023; Muner et al., 2024; Sun et al., 2024; Tang et al., 2024). For hv*Kp* isolates in particular, more and more genetic factors are being described that lead to attenuated capsule synthesis compared to other pathotypes and offer a survival advantage in the host organism (Walker et al., 2020). In particular, the presence of the transcriptional regulators RmpA and RmpA2 is associated with a hypermucoviscous phenotype and increased virulence (Lai et al., 2003; Cheng et al., 2010; Hsu et al., 2015; Lin et al., 2020; Chu et al., 2023).

Just recently, Russo et al. published a study where they investigated 49 K. *pneumoniae* strains possessing varying combinations of siderophore and other virulence genes associated with a hypervirulence phenotype and with different antibiotic resistance properties (Russo et al., 2024). Altogether 16 isolates were categorized as hv*Kp* and 33 as c*Kp* based on their behavior in a murine infection model. Biomarker presence, siderophore production, mucoviscosity, virulence plasmid homogeneities, and Kleborate virulence scores were measured and evaluated to accurately differentiate pathotypes. The presence of all the investigated biomarkers *iucA*, *iroB*, peg-344, *rmpA*, and *rmpA2* was most accurate (94%) to predict hypervirulence; whereas the presence of ≥4 of these biomarkers was most sensitive (100%). Even when using such an extensive and well-defined strain collection and informative models and logistic regressions, predictions of hypervirulence in *K. pneumoniae* isolates remains challenging.

In a recent paper, Masson et al., describe that *K. pneumoniae* O1 antigen prevents complement mediated killing when these isolates were compared to a larger collection of *K. pneumoniae* isolates with other O antigens (Masson et al., 2024). We did not see any link between pathotypes, presence of O antigens and growth and survival in serum samples or platelet concentrates (**Figures 8**, **9**). Admittedly, our strain collection was less variable in terms of O-antigen structure. However, *K. pneumoniae* isolates with identical O1-antigens behaved differently in the two mentioned experimental settings suggesting the important role of components other than the O locus involved in growth and survival in blood or similar compartments.

### Limitations of the study

Our study has several limitations. First, the classification into the three groups c*Kp*, ESBL-c*Kp* and hv*Kp* is oversimplified because it ignores major differences in genome content, in addition to the group designations, between the isolates within a given group and across different categories. These differences may potentially also influence cell-biological behavior in the given experiments. Second, we might have validated our experimental findings by including more than two isolates per given group. The limitation in isolate number per group is due to the fact that each experiment had been executed three times at least due to statistical validations, and that we included quite a high number of different adhesion, invasion and replication experiments and various cell line types. We have extensively tested and analyzed a larger set of cell lines and experimental settings beforehand, which is not described in greater details in this manuscript. Third, we are well aware that using isogenic strains would be beneficial for the experimental settings. For instance, using two strain types just differing by a *bla*_ESBL_-containing plasmid (e.g., as a c*Kp* vs. ESBL-c*Kp*) would better specify some findings and let assume to point towards physiological and immunological behavior to a few genomic differences only. However, well-defined and characterized *K. pneumoniae* recipients were not available when we planned and started our experiments some years ago. Additionally, genetic strategies to cure plasmids safely from *K. pneumoniae* strains were not established. Forth, it would have been advantageous, if we have used a complete and entire strain set through all the experiments included. The selection and limitation to a smaller or slightly different isolate selection was due to organizational and infrastructural requirements and demands of QM/QC at the different research institutions involved.

## Conclusions

In summary, the data from the *in vitro* infection experiments showed that *K. pneumoniae* isolates representing various pathotypes (c*Kp*, ESBL-c*Kp*, hv*Kp*) differ in the host-pathogen interactions with regard to their invasion, resistance to phagocytosis and replication potential in macrophages. Thus, evidence was found that the successful spread of strains representing distinct *K. pneumoniae* lineages is not solely due to a survival advantage under selective pressure by antibiotics, which contributes to a better understanding of pathogenic mechanisms of *K. pneumoniae*. A stronger cytotoxic effect on intestinal cells was observed for hv*Kp*, which presumably favors their translocation. Furthermore, it could be shown that the pure presence of siderophore genes have no positive influence on the proliferation of *K. pneumoniae* in blood.

## Data Availability

The data presented in the study are deposited in the ENA repository, project accession number PRJEB85663.
